# Co-existence of acute myeloid leukemia with multilineage dysplasia and Epstein-Barr virus-associated T-cell lymphoproliferative disorder in a patient with rheumatoid arthritis: a case report

**DOI:** 10.1186/1756-8722-2-27

**Published:** 2009-06-30

**Authors:** Michihide Tokuhira, Kyoko Hanzawa, Reiko Watanabe, Yasunobu Sekiguchi, Tomoe Nemoto, Yasuo Toyozumi, Jun-ichi Tamaru, Shinji Itoyama, Katsuya Suzuki, Hideto Kameda, Shigehisa Mori, Masahiro Kizaki

**Affiliations:** 1 Division of Hematology, Saitama Medical Center, Saitama Medical University, Kawagoe, Saitama, Japan; 2 Division of Pathology, Saitama Medical Center, Saitama Medical University, Kawagoe, Saitama, Japan; 3 Department of Rheumatology, Saitama Medical Center, Saitama Medical University, Kawagoe, Saitama, Japan

## Abstract

Rheumatoid arthritis (RA) is an autoimmune disease mediated by inflammatory processes mainly at the joints. Recently, awareness of Epstein-Barr virus (EBV)-associated T-cell lymphoproliferative disorder (T-LPD) has been heightened for its association with methotraxate usage in RA patients. In the contrary, acute myeloid leukemia with multilineage dysplasia (AML-MLD) has never been documented to be present concomitantly with the above two conditions. In this report we present a case of an autopsy-proven co-existence of AML-MLD and EBV-associated T-LPD in a patient with RA.

## Background

Rheumatoid arthritis (RA) is an autoimmune disease mediated by inflammatory processes mainly at the joints; activation of fibroblasts and macrophages of the synovial tissue by a triggering agent(s) is thought to play a role in its pathogenesis, while lymphocytes in these environments may play an important role in the destruction of joint tissue by the RA-associated autoimmunity [1-3]. In the present case, two additional diseases, i.e., acute myeloid leukemia with multilineage dysplasia (AML-MLD) and Epstein-Barr virus (EBV)-associated T-cell lymphoproliferative disorder (T-LPD), developed after the treatment of RA. The patient died with respiratory complications and multiple organ failure with severe infection after steroid pulse therapy and cyclophosphamide. To the best of our knowledge, this is the first report of the simultaneous presence of AML-MLD and EBV-associated T-LPD in a patient with RA.

## Case report

**Figure 1:**
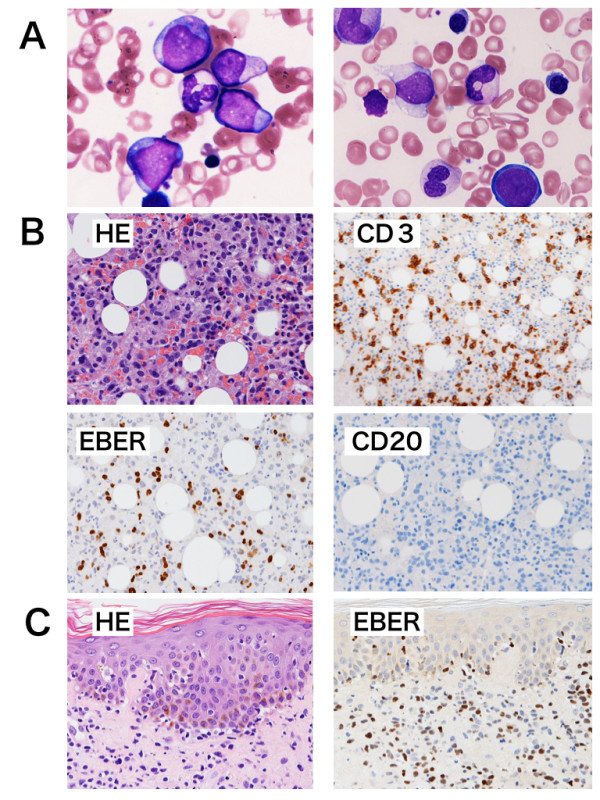
**A: Morphology of bone marrow (BM) aspiration**. The BM is hypoplastic, with increased myeloid blasts. The erythroid series showed dysplastic changes (Wright-May-Giemsa staining; original magnification: ×800). B and C: Hematoxylin and eosin (HE) staining and double immunohistochemistry analyses of BM biopsy (B) and skin biopsy (C). B: HE showed the diffuse infiltration of small lymphocytes into BM tissue, and these cells expressed CD3, but not CD20. Furthermore, EBER in situ hybridization analyses was positive. (original magnification: ×400) C: Small lymphocytes invaded the dermal lesion, especially around capillary blood vessels, and the phenotype of these cells was similar to those seen in BM. (original magnification: ×400)

**Figure 2:**
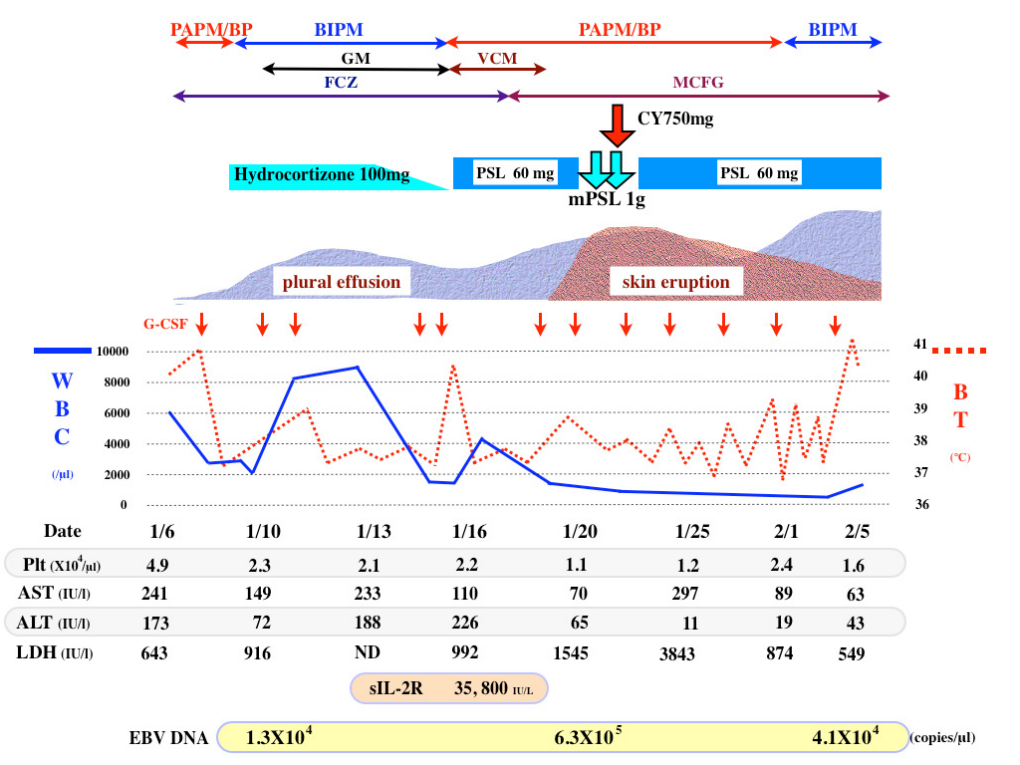
**A Diagraph of the clinical course**. Detailed description was provided in the case report. PAPM/BP: panipenem/betamiprom; BIPM: biapenem; GM: gentamicin; VCM: vancomycin; FCZ: fluconazole; MCFG: micafungin; PSL: prednisolone; mPSL: methylprednisolone sodium succinate; CY: cyclophosphamide; WBC: white blood cell; BT: body temperature; Plt: platelet; Fib: fibrinogen; AST: aspartate aminotransferase; ALT: alanine aminotransferase; LDH: lactate dehydrogenase; sIL-2: soluble interleukin-2 receptor; ND: not done.

**Figure 3:**
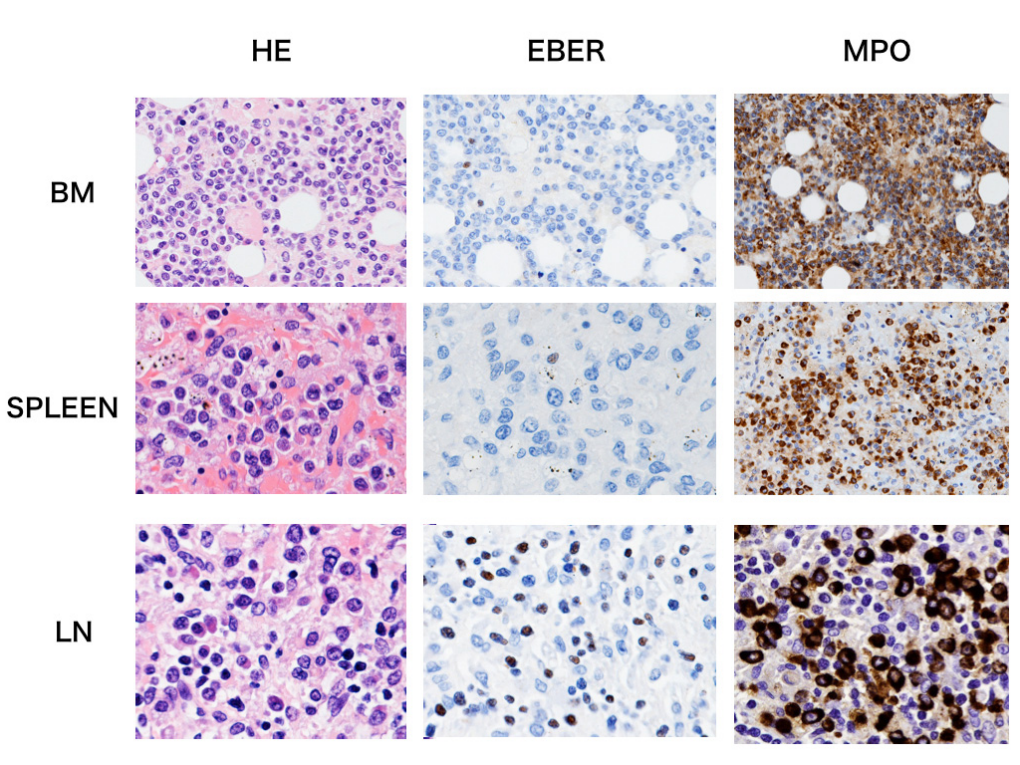
**Hematoxylin and eosin (HE) staining and immunohistochemistry analyses of bone marrow (BM), spleen, and lymph nodes (LNs) from autopsy**. HE staining of BM showed hypercellularity, and increased blasts. These blasts expressed MPO, whereas the population of lymphocytes was small and there was almost no staining for EBER in the BM. The spleen tissue also showed diffuse infiltration of myeloblasts, and rare EBER-positive lymphocytes. In contrast, leukemic cells and EBER-positive lymphocytes were seen in the LNs. (magnification: ×800).

A 64-year-old previously healthy man visited our hospital with arthralgia and morning stiffness in July 2005. Physical examination revealed no dry eye, dry mouth, erythematous nodules or other autoimmune-mediated manifestations. His blood test results were unremarkable; his white blood cell (WBC), red blood cell, and platelet counts were normal, and C-reactive protein (CRP) was negative. Serological examination indicated positivity for anti-nuclear antibody (ANA) (1:80, speckled pattern), and negativity for anti-double strand DNA antibody, rheumatoid factor, anti-RNP antibody, and anti-SS-A antibody. Bilateral hand X-rays showed mild swelling and destruction of the metacarpo-phalangeal (MP) and proximal-inter-phalangeal (PIP) joints. He was diagnosed to have RA. He had unsatisfactory response to anti-non steroid inflammatory drugs (NSAIDS). We therefore administered prednisolone (PSL; 5 mg/day) and bucillamine (200 mg/day), but discontinued the bucillamine after 4 months due to skin rash and eye lid edema. His regular blood tests revealed worsening anemia and thrombocytopenia, and he was admitted to our hospital for further examination. Blood examination revealed mild leukocytosis (9,600/cu mm) with increase of blasts (43%), anemia (Hg 7.9 g/100 mL) and thrombocytopenia (3.1 × 10^4^/cu mm). Blood biochemical examination disclosed slight elevation of the serum levels of lactate dehydrogenase (LDH: 304 IU/mL). Bone marrow (BM) examination revealed an increase of myeloid blasts (23.1%) with dysplasia in three myeloid cell lineages (Figure 1A), and a diagnosis of AML-MLD was made based on World Health Organization (WHO) criteria [4]. The immunophenotype of blasts was CD7, 13, 33, 34, and HLA-DR positive, and an abnormal karyotype, i(7)(p10), was detected in 7 of 20 cells examined. After receiving two courses of low-dose Ara-C (30 mg continuous intravenous drip injection (d.i.v.) for 14 days), the patient achieved partial remission, and additional chemotherapy consisting of two courses of CAG (Ara-C 30 mg/day continuous d.i.v. for 14 days, aclarubicin 10 mg/day on days 1–3, and G-CSF 250 mcg d.i.v. on days 1–14) led to complete remission (CR). During the chemotherapy, RA manifestations such as arthralgia and morning stiffness were not observed. After achieving CR, he remained well for several weeks as an outpatient, but high fever and dyspnea suddenly appeared in January 2007. He was admitted again, and antibiotics and anti-fungal drugs were administered with no improvement. His blood test indicated pancytopenia (WBC: 1,700/cu mm; Hb: 12.4 g/100 mL; platelets: 5.1 × 10^4^/cu mm), liver dysfunction (aspartate aminotransferase (AST): 100 IU/L; alanine aminotransferase (ALT): 78 IU/L), and elevated LDH (525 IU/L). His WBC showed relative neutropenia, and monocytosis without blast cell increase. His CRP was high (4.0 mg/100 mL), and his ferritin level was extremely elevated (46,802 ng/mL). Antibodies directed against cytomegalovirus (CMV), human T-cell lymphotropic virus type 1 (HTLV-1) and human immunodeficiency virus (HIV) I/II were negative. The EBV serology of this patient revealed an existing infectious pattern, i.e., anti-Epstein-Barr virus-viral capsid antigen (VCA) IgM <10, anti-VCA IgG X9.5, and anti-Epstein-Barr virus nuclear antigen (EBNA) X6. The aPTT and PT were prolonged (54% and 53 seconds, respectively), and FDP (52 pg/100 mL) was elevated, suggesting DIC state. Chest X-ray indicated mild cardiomegaly with massive pleural effusion, and whole body computer tomography showed other abnormalities, such as ascites, axillary and para aorta lymphadenopathies and splenomegaly (not shown). The level of soluble interleukin-2 receptor in serum was extremely elevated (35,800 IU/L). A thoracentesis was done to evaluate the etiology. Cytology study indicated no malignancy and culture revealed no bacterial/fungal/tubercular infection. Therefore, hydrocortisone was given to relieve the symptoms. The clinical course was shown in Figure 2. A BM examination showed residual blast cells, but small lymphocytes were increased, expressing the CD3+CD4-CD8-CD19-CD20-CD56-MPO-phenotype, and EBER was also detected (Figure 1B). Based on these facts, EBV-mediated T-LPD was diagnosed. PSL (60 mg/day) was started. Although fever, pleural effusion and liver dysfunction showed a partial response to this medication, the skin rash and LDH elevation progressed (Figure 2). A skin biopsy was performed and revealed T-cell infiltration in the dermal lesion, with phenotype similar to that seen in BM tissue (Figure 1C). This suggested persistent T-LPD. Thus high dose steroid pulse therapy (methylprednisolone 1 g/day for 2 days) and cyclophosphamide (750 mg d.i.v. for a day) were administered. However, patient's condition worsened rapidly. Three weeks after the diagnosis of T-LPD, the patient died of multiple organ failure with pneumonia and sepsis. An autopsy revealed the presence of leukemic cell infiltration into multiple organs: the BM, liver, spleen, lymph nodes (LNs), pancreas, and adrenal glands (Figure 3). In addition, EBER was negative in all these organs except the LNs. Both myeloid blasts and EBER-positive small T-lymphocytes were detected in the LNs (Figure 3). The lung tissue did not show infiltration of AML cells or EBV-infected T cells; however, gram-negative bacteria, aspergillus and mucor infection were detected. Moreover, massive alveolar bleeding and congestion were also documented. The finger joints were slightly deformed, and the membranes of these joints showed mild synovial and lymphoid proliferation. These findings were compatible with the pathological findings of RA joints.

## Discussion

To the best of our knowledge, this is the first reported case of co-existing AML-MLD and EBV-associated T-LPD in RA. It is possible that the development of AML was secondary to either RA-related treatment or the underlying myelodysplastic syndrome (MDS) [5-8]. In the present case, NSAIDs, PSL and bucillamine were given to the patient as RA treatment. Bucillamine was developed in Japan, and has a cysteine derivative possessing two SH-groups. Its antirheumatic effects are thought to arise from its suppression of the formation of IgM in B cells, the formation of matrix metalloproteinase-3, and the differentiation of osteoclasts [9,10]. The bone marrow karyotyping revealed an abnormal karyotype of chromosome 7 in the BM cells, which is seen often in MDS. It is therefore most likely that AML-MLD was secondary to MDS. It has been shown that EBV infection can trigger chronic immune inflammatory disease [11,12]. For instance, the number of infected peripheral B lymphocytes in RA tend to be higher than in normal individuals, and an impairment of specific cytotoxic T lymphocytes against EBV has been noted in RA patients [13,14]. In addition, EBV DNA was directly detected in RA synovial tissue by polymerase chain reaction method [15]. Balandraud et al demonstrated that RA has a 10-fold systemic EBV overload, very similar to that observed in organ transplant recipients [16]. In the present case, high titers of EBV were seen. Recent attention has been focused on this immunodysregulatory phenomena. It has been demonstrated that the responsible gene is SAP (signaling lymphocytic activation molecule [SLAM]-associated protein), an adaptor protein that mediates signals through SLAM and other immunoglobulin superfamily receptors including 2B4, Ly8, SF2000, and CD84 [17]. It has been suggested that SAP plays an important role in the physiological immunity for viral infections [18,19]. In regard to RA patients, Takei et al demonstrated that the expression level of SAP transcripts in the peripheral leukocytes of RA patients was significantly lower than in normal individuals, and RA patients had decreased expression of SAP transcripts in peripheral CD2(+) T cells compared to normal individuals. They proposed that decreased SAP gene expression might trigger RA progression [20,21]. On the other hand, EBV-LPD has often been reported in immunodeficient individuals such as HIV patients, patients post-transplantation, or patients taking immunosuppressants [22]. Methotrexate (MTX) has been implicated to induce LPD in RA patients [23]. The fact that withdrawal of MTX led to improvement of LPD in 30–50% of the patients also suggested a direct MTX interaction with immune system [24]. Several studies have reported that RA itself is not a risk factor of LPD [25]. It remains unclear whether the co-existence of the three conditions are coincidental or there could be an intrinsic mechanism.

## Conclusion

The current case with AML-MLD and EBV-associated T-LPD developmedin a RA patient appears to be extremely rare. To the best of our knowledge, this is the first reported case of co-existing AML-MLD and EBV-associated T-LPD in a patient with RA.

## Abbreviations

RA: rheumatoid arthritis; AML-MLD: acute myeloid leukemia with multilineage dysplasia; EBV: Epstein-Barr virus; T-LPD: T-cell lymphoproliferative disorder; WBC: white blood cell; CRP: C-reactive protein; ANA: anti-nuclear antibody; NSAIDs: anti-non steroid inflammatory drugs; PSL: prednisolone; LDH: lactate dehydrogenase; BM: bone marrow; WHO: World Health Organization; CR: complete remission; AST: aspartate aminotransferase; ALT: alanine aminotransferase; CMV: cytomegalovirus; HTLV-1: human T-cell lymphotropic virus type 1; HIV: human immunodeficiency virus; VCA: anti-Epstein-Barr virus-viral capsid antigen; EBNA: anti-Epstein-Barr virus nuclear antigen; LN: lymph node; MDS: myelodysplastic syndrome; MTX: methothraxate; SAP: signaling lymphocytic activation molecule associated protein; SLAM: signaling lymphocytic activation molecule.

## Consent

Written informed consent was obtained from the patient's wife for publication of this case report and any accompanying images. A copy of the written consent is available for review by the Editor-in-Chief of this journal.

## Competing interests

The authors declare that they have no competing interests.

## Authors' contributions

MT, KS and JT assembled, analyzed and interpreted the patient findings including the hematological disease, rheumatoid arthritis and pathological samples. All authors contributed to writing the manuscript. All authors read and approved the final manuscript.

## References

[B1] American College of Rheumatology Subcommittee on Rheumatoid Arthritis Guidelines (2002). Guidelines for the management of rheumatoid arthritis: 2002 Update. Arthritis Rheum.

[B2] Huber LC, Stanczyk J, Jungel A, Gay S (2007). Epigenetics in inflammatory rheumatic diseases. Arthritis Rheum.

[B3] Visser H, le Cessie S, Vos K, Breedveld FC, Hazes JM (2002). How to diagnose rheumatoid arthritis early: a prediction model for persistent (erosive) arthritis. Arthritis Rheum.

[B4] Brunning RD (2003). Classification of acute leukemias. Semin Diagn Pathol.

[B5] Singh ZN, Huo D, Anastasi J, Smith SM, Karrison T, Le Beau MM, Larson RA, Vardiman JW (2007). Therapy-related myelodysplastic syndrome: morphologic subclassification may not be clinically relevant. Am J Clin Pathol.

[B6] Mauritzson N, Albin M, Rylander L, Billström R, Ahlgren T, Mikoczy Z, Björk J, Strömberg U, Nilsson PG, Mitelman F, Hagmar L, Johansson B (2002). Pooled analysis of clinical and cytogenetic features in treatment-related and de novo adult acute myeloid leukemia and myelodysplastic syndromes based on a consecutive series of 761 patients analyzed 1976–1993 and on 5098 unselected cases reported in the literature 1974–2001. Leukemia.

[B7] Rosenthal NS, Farhi DC (1996). Myelodysplastic syndromes and acute myeloid leukemia in connective tissue disease after single-agent chemotherapy. Am J Clin Pathol.

[B8] Okamoto H, Teramura M, Kamatani N (2004). Myelodysplastic syndrome associated with low-dose methotrexate in rheumatoid arthritis. Ann Pharmacother.

[B9] Okazaki H, Sato H, Kamimura T, Hirata D, Iwamoto M, Yoshio T, Mimori A, Masuyama JI, Kano S, Minota S (2000). In vitro and in vivo inhibition of activation induced T cell apoptosis by bucillamine. J Rheumatol.

[B10] Munakata Y, Iwata S, Dobers J, Ishii T, Nori M, Tanaka H, Morimoto C (2000). Novel in vitro effects of bucillamine: inhibitory effects on proinflammatory cytokine production and transendothelial migration of T cells. Arthritis Rheum.

[B11] Balandraud N, Roudier J, Roudier C (2004). Epstein-Barr virus and rheumatoid arthritis. Autoimmun Rev.

[B12] Callan MF (2004). Epstein-Barr virus, arthritis, and the development of lymphoma in arthritis patients. Curr Opin Rheumatol.

[B13] Tosato G, Steinberg AD, Yarchoan R, Heilman CA, Pike SE, De Seau V, Blaese RM (1984). Abnormally elevated frequency of Epstein-Barr virus-infected B cells in the blood of patients with rheumatoid arthritis. J Clin Invest.

[B14] Tosato G, Steinberg AD, Blaese RM (1981). Defective EBV-specific suppressor T-cell function in rheumatoid arthritis. N Engl J Med.

[B15] Saal JG, Krimmel M, Steidle M, Gerneth F, Wagner S, Fritz P, Koch S, Zacher J, Sell S, Einsele H, Müller CA (1999). Synovial Epstein-Barr virus infection increases the risk of rheumatoid arthritis in individuals with the shared HLA-DR4 epitope. Arthritis Rheum.

[B16] Balandraud N, Meynard JB, Auger I, Sovran H, Mugnier B, Reviron D, Roudier J, Roudier C (2003). Epstein-Barr virus load in the peripheral blood of patients with rheumatoid arthritis: accurate quantification using real-time polymerase chain reaction. Arthritis Rheum.

[B17] Yin L, Al-Alem U, Liang J, Tong WM, Li C, Badiali M, Médard JJ, Sumegi J, Wang ZQ, Romeo G (2003). Mice deficient in the X-linked lymphoproliferative disease gene sap exhibit increased susceptibility to murine gammaherpesvirus-68 and hypo-gammaglobulinemia. J Med Virol.

[B18] Sawada S, Takei M, Ishiwata T (2007). SAP discovery: the sword edges-beneficial and harmful. Autoimmun Rev.

[B19] Wu C, Nguyen KB, Pien GC, Wang N, Gullo C, Howie D, Sosa MR, Edwards MJ, Borrow P, Satoskar AR, Sharpe AH, Biron CA, Terhorst C (2001). SAP controls T cell responses to virus and terminal differentiation of TH2 cells. Nat Immunol.

[B20] Takei M, Ishiwata T, Mitamura K, Fujiwara S, Sasaki K, Nishi T, Kuga T, Ookubo T, Horie T, Ryu J, Ohi H, Sawada S (2001). Decreased expression of signaling lymphocytic-activation molecule-associated protein (SAP) transcripts in T cells from patients with rheumatoid arthritis. Int Immunol.

[B21] Sawada S, Takei M, Inomata H, Nozaki T, Shiraiwa H (2007). What is after cytokine-blocking therapy, a novel therapeutic target – synovial Epstein-Barr virus for rheumatoid arthritis. Autoimmun Rev.

[B22] Rezk SA, Weiss LM (2007). Epstein-Barr virus-associated lymphoproliferative disorders. Hum Pathol.

[B23] Hoshida Y, Xu JX, Fujita S, Nakamichi I, Ikeda J, Tomita Y, Nakatsuka S, Tamaru J, Iizuka A, Takeuchi T, Aozasa K (2007). Lymphoproliferative disorders in rheumatoid arthritis: clinicopathological analysis of 76 cases in relation to methotrexate medication. J Rheumatol.

[B24] Miyazaki T, Fujimaki K, Shirasugi Y, Yoshiba F, Ohsaka M, Miyazaki K, Yamazaki E, Sakai R, Tamaru J, Kishi K, Kanamori H, Higashihara M, Hotta T, Ishigatsubo Y (2007). Remission of lymphoma after withdrawal of methotrexate in rheumatoid arthritis: relationship with type of latent Epstein-Barr virus infection. Am J Hematol.

[B25] Kamel OW, Holly EA, Rijn M van de, Lele C, Sah A (1999). A population based, case control study of non-Hodgkin's lymphoma in patients with rheumatoid arthritis. J Rheumatol.

